# Prebiotic Effects and Fermentation Kinetics of Wheat Dextrin and Partially Hydrolyzed Guar Gum in an *In Vitro* Batch Fermentation System

**DOI:** 10.3390/foods4030349

**Published:** 2015-08-21

**Authors:** Justin Carlson, Ashok Hospattankar, Ping Deng, Kelly Swanson, Joanne Slavin

**Affiliations:** 1 Department of Food Science and Nutrition, University of Minnesota—Twin Cities 1334 Eckles Ave St. Paul, MN 55108, USA; E-Mail: carl2814@umn.edu; 2 Novartis Consumer Health, Inc. 200 Kimball Drive Parsippany, NJ 07054-0622, USA; E-Mail: ashok.v.hospattankar@gsk.com; 3 Department of Animal Sciences and Division of Nutritional Sciences, University of Illinois—Urbana-Champaign 1207 West Gregory Drive Urbana, IL 61801, USA; E-Mails: deng12@illinois.edu (P.D.); ksswanso@illinois.edu (K.S.)

**Keywords:** SCFA, butyrate, *Lactobacillus*, *Bifidobacterium*, prebiotic

## Abstract

Scientific research demonstrates that two indigenous gut bacteria, *Lactobacillus* and *Bifidobacterium* can contribute to human health. Although these bacteria can be consumed as probiotics, they can also be produced in the gut by bacteria, and are then called prebiotics. The primary objective of this *in vitro* study was to quantitatively analyze at the genus level how two dietary fibers, wheat dextrin (WD) and partially hydrolyzed guar gum (PHGG) changed the levels of these two gut bacteria at 12 and 24 h, via real time qualitative polymerase chain reaction (qPCR). Secondary objectives were changes in fecal pH, short chain fatty acids (SCFAs) and total gas volume produced. At 12 h WD was more bifidogenic (9.50 CFU log_10_/mL) than PHGG (9.30 CFU log_10_/mL) (*p* = 0.052), and also at 24 h WD (9.41 CFU log_10_/mL) compared with PHGG (9.27 CFU log_10_/mL) (*p* = 0.043). WD produced less total SCFAs at both 12 and 24 h than PHGG, and produced significantly lower amounts of gas at 12 and 24 h (*p* < 0.001). Both PHGG and WD also promoted growth of *Lactobacilli* when measured at 12 and 24 h compared with the 0 h analysis, indicating that both fibers are lactogenic. These results demonstrate the prebiotic effect of WD and PHGG. Based on fermentation kinetics, PHGG is more rapidly fermented than WD, and both fibers show prebiotic effects as early as 12 h.

## 1. Introduction

Dietary fiber is generally defined as nondigestible carbohydrates and lignin that are intrinsic and intact in plants, with functional fiber showing beneficial physiological effects in humans [1]. Currently the U.S. recommendations for dietary fiber, expressed as a Dietary Reference Intake, are 25 g/day for adult females and 38 g/day for adult males, although the typical daily intake for U.S. consumers is approximately 17 g/day [2,3]. Generally accepted health benefits associated with regular fiber consumption include maintaining a healthy digestive system, increased satiety, decreased caloric intake, and fermentation that results in beneficial changes in the gut microflora [4,5]. Daily supplementation of beneficial dietary fibers may be an effective way to help consumers get the recommended amount of dietary fiber, and its associated health benefits.

Wheat dextrin (WD) and partially hydrolyzed guar gum (PHGG) are dietary fibers evaluated in this study. WD is a soluble, fermentable fiber composed of a glucose polymer formed by the polymerization and hydrolysis of wheat starch that resists digestion in the small intestine due to its glucoside linkages [6]. PHGG is a hydrolyzed product of guar gum, composed of mannose and galactose monomers. Clinical studies have shown that 45 g/day of WD is well tolerated in the gastrointestinal tract [7]. WD also has been shown to decrease hunger and increase satiety in a randomized, double-blind, placebo-controlled study [8,9]. PHGG has been shown in randomized, cross-over clinical studies to reduce hunger and increase satiety [10,11].

Two different soluble fibers were selected in this study to help understand the mechanisms of action and subsequent changes in gut microflora through the use of an *in vitro* batch fermentation model. *In vitro* fermentation methods are a representative model of colonic fermentation, and work well to predict substrate availability and ability for fermentation in the gut [12]. Previous preliminary studies have shown that WD and PHGG ferment in the large intestine producing measurable levels of beneficial *Bifidobacteria* and *Lactobacillus* at 24 h, indicating that both dietary fibers demonstrate a prebiotic effect [13].

The primary objective of this study was to measure changes in two beneficial genera of microbes, *Lactobacillus* and *Bifidobacterium*, to better understand the prebiotic effects of WD and PHGG and their mechanisms of action. Secondary objectives included measurements of common fermentation markers, such as pH, total gas volume and short-chain fatty acids (SCFA).

## 2. Materials and Methods

Fibers investigated in this study included wheat dextrin (Benefiber^®^, Novartis Consumer Health Inc., Parsippany, NJ, USA) and partially hydrolyzed guar gum (Benefibra™, Novartis Consumer Health Spa Origgio, Varese, Lombardy, Italy). A substrate blank was employed for all baseline measurements in fecal inoculum. Chemical reagents used in this study were provided by ThermoFisher Scientific (ThermoFisher Scientific Inc., Waltham, MN, USA), Sigma-Aldrich (Sigma-Aldrich, St. Louis, MO, USA) and Oxyrase (Oxyrase Inc., Mansfield, OH, USA).

### 2.1. Fecal Collection

Fecal samples were collected from three healthy volunteers (2 males, 1 female) under anaerobic conditions from three individuals (ages 18–28) consuming non-specific Western diets, free of antibiotic treatments in the last 6 months, not affected by any GI diseases and not consuming any prebiotic or probiotic supplements. Fecal samples were anaerobically collected within 1 h of the start of the batch fermentation and homogenized immediately upon collection.

### 2.2. Fermentation

Fiber samples (0.5 g) were hydrated in 40 mL of prepared sterile tricase peptone fermentation media in 100 mL serum bottles, capped to avoid contamination, and incubated for 12 h at 4 °C. Following incubation, serum bottles were transferred to a circulating water bath at 37 °C and allowed to incubate for 2 h. Post-collection, fecal samples were homogenized using a 6:1 ratio of phosphate buffer solution to fecal matter. After mixing, obtained fecal slurry was combined with prepared reduction solution (2.52 g cysteine hydrochloride, 16 mL 1N NaOH, 2.56 g sodium sulfide nonanhydride, 380 mL DD H_2_O) at a 2:15 ratio. 10 mL of the prepared fecal inoculum was added to each of the serum bottles, 0.8 mL Oxyrase^®^ added, flushed with CO_2_, sealed, and then immediately placed in the 37 °C circulating water bath. Samples were prepared in triplicate and analyzed at 0, 4, 8, 12 and 24 h. Triplicate positive controls (dextrose) and negative controls (substrate blank) were also analyzed at the same time points. Upon removal at appropriate time point, pH and total gas volume were measured. Then, 1 mL of copper sulfate (200 g/L) was added to cease fermentation. Lastly, 2 mL aliquots were frozen at −80 °C for further analysis.

### 2.3. pH Analysis

Two milliliter aliquots were removed from serum bottles immediately following total gas measurement and measured with an Orion PerpHect LogR Meter—Model 350 (Orion Research, Inc., Boston, MA, USA).

### 2.4. Gas Analysis

Total gas production was measured by syringe difference analysis. Gas was measured by piercing cap of serum bottle with syringe needle and measuring gas released from system.

### 2.5. SCFA Analysis

SCFA extraction methods were adapted and modified from Schneider *et al.* [14] 2 mL aliquots were removed from the −80 °C freezer and placed in 4 °C cooler for 12 h to thaw prior to SCFA analysis. Tubes were then gently vortexed for 5 s. Then, 1.6 mL of DI H_2_0, 400 μL H_2_SO_4_ (50% *vol/vol*), and 2 mL diethyl ether (premixed with 2-ethyl butyric acid as internal standard) were all added to tubes and vortexed again for 5 s. Tubes were then placed in an orbital shaker for 45 min at 100 RPM. Tubes were removed and then centrifuged for 5 min at 3000 RPM. Supernatant was removed from tube and placed in 10 mL plastic tubes containing CaCl_2_ to remove any residual water. Solution was then filtered using a BD 1 mL syringe (Becton, Dickinson and Company Franklin Lakes, NJ, USA) and a Millex 13 mm nylon membrane filter with a 0.20 μm pore size (Merck Millipore Ltd Tullagreen, Carrigtwohill, Co. Cork, Ireland). Extractions were then analyzed using a HP 5890 series gas chromatograph (Hewlitt Packard, Palo Alto, CA, USA) with a 30 m × 0.250 mm × 0.25 μm polyethylene glycol (PEG) column (Agilent Technologies, Santa Clara, CA, USA), with a 110 °C oven temperature. Samples were injected using an automated HP 7673 GC/SFC injector (Hewlitt Packard, Palo Alto, CA, USA). Injector and detector temperatures were 220 and 240 °C, respectively. Flow rates for air, helium and hydrogen were 26, 28 and 315 mL/min, respectively. All samples were analyzed with a 50:1 split ratio.

### 2.6. Microbiota Analysis—Quantitative Polymerase Chain Reaction (qPCR)

*Bifidobacterium* genus and *Lactobacillus* genus were quantified by DNA extraction from fermented samples, followed by qPCR using specific primers. Amplification was performed in a set of triplicate reactions for each bacterial group within each sample according to the procedures of Hernot *et al.* [15] For amplification, 10 μL final volume containing 2X SYBR Green PCR Master Mix (Applied BioSystems, Foster City, CA, USA), 15 pmol of each primer, and 10 ng of template DNA were used. Standard curves were obtained by harvesting pure cultures of each bacterium in the logarithmic growth phase in triplicate to create a five-fold dilution series. DNA from each serial dilution was extracted using a PowerSoil DNA Isolation Kit (MO BIO Laboratories, Inc., Carlsbad, CA, USA) and amplified along with fecal DNA samples using a Taqman ABI PRISM 7900HT Sequence Detection System (Applied BioSystems). The colony forming units (CFU) of each standard curve serial dilution was determined by plating the *Lactobacillus* genus on Difco *Lactobacilli* MRS broth (Becton, Dickenson, and Company, Sparks, MD, USA), and *Bifidobacterium* genus on Difco Reinforced Clostridial Medium (Becton, Dickenson, and Company, Sparks, MD, USA). Cycle threshold (Ct) values were plotted against standard curves for quantification (CFU/mL) of the target bacterial DNA from fermentation samples.

### 2.7. Statistical Analysis

Statistical analysis was conducted using SAS statistical program software version 9.3 (SAS Institute, Cary, NC, USA). Analysis of variance (ANOVA) with Tukey pair-wise was used for all tests measuring differences of means. Statistical significance was achieved for *p*-values less than 0.05. Log transformations were implemented as needed for analysis.

## 3. Results

### 3.1. Gas Production and pH Shift

**Figure 1:**
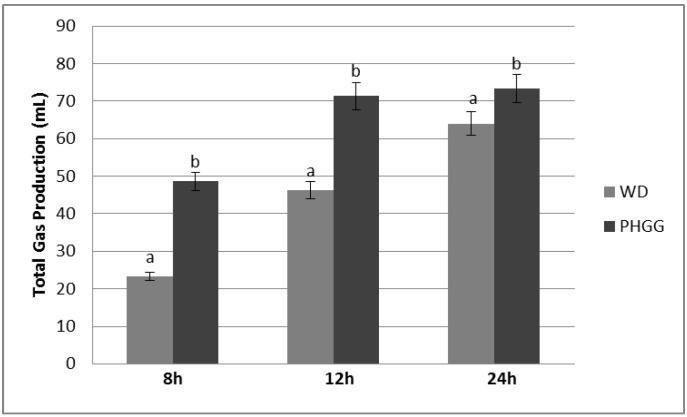
Total Gas Volume Produced in Batch Culture Fermentation System with Wheat Dextrin (WD) and Partially Hydrolyzed Guar Gum (PHGG) Across 24 h of Analysis. Notes: Columns with different letters are significantly different from one another within the same time of measurement (*p* < 0.05), at 8, 12 and 24 h; Values displayed are mean values (*n* = 3) ± SE; No measurable amounts of gas were detected in the substrate blank, or at 0 or 4 h for WH and PHGG.

Within the first four hours of analysis, neither WD nor PHGG produced detectable amounts of gas. At 8 h, PHGG produced significantly more gas (*p* < 0.001) than WD, as well as at 12 and 24 h of fermentation (Figure 1). Each fiber produced a significant decrease in pH (Table 1), with WD decreasing more consistently over 24 h, while PHGG decreased quickly between 4 and 8 h of fermentation, which is simultaneously reflected by the large increase in gas production at 8 h for PHGG.

**Table 1:** pH Shift in Batch Culture Fermentation System with WD and PHGG Across 24 h of Analysis.

Time (h)	pH		
	WD	PHGG	Blank
0	6.96 (0.02) a	6.97 (0.01) a	7.06 (0.01) b
4	6.51 (0.00) a	6.59 (0.02) b	6.96 (0.01) c
8	6.34 (0.00) b	5.93 (0.00) a	6.92 (0.00) c
12	6.07 (0.01) b	5.90 (0.00) a	6.92 (0.01) c
24	5.78 (0.02) a	5.86 (0.00) b	6.92 (0.01) c

Notes: Values are mean (*n* = 3) followed by (SE); Values with different letters are statistically different from each other within rows (*p* < 0.05).

### 3.2. SCFA Production

Levels of acetate, propionate and butyrate indicate that increased amounts of SCFAs can be seen with time in the batch culture fermentation system for both WD and PHGG. Levels for PHGG remained constant or declined after 12 h. WD produced less total SCFA (data not shown) compared to PHGG at 12 h (*p* = 0.015). All levels expressed (Table 2) are expressed as μmol SCFA/mL of system media.

**Table 2:** Short Chain Fatty Acids (SCFAs) of Exposed Human Fecal Inoculum across 24 h of Analysis.

Time (h)	Acetate (μmol/mL)		Propionate (μmol/mL)		Butyrate (μmol/mL)	
	WD	PHGG	WD	PHGG	WD	PHGG
0	1.20 (0.05)	1.03 (0.09)	0.69 (0.01)	0.68 (0.02)	0.98 (0.03)	0.95 (0.03)
4	5.02 (0.38)	4.30 (0.14)	2.45 (0.17)	2.36 (0.03)	3.70 (0.29)	3.22 (0.05)
8	7.56 (0.36)	9.83(0.01)	7.37 (0.19)	7.47 (0.29)	7.77 (0.39)	10.48 (0.77)
12	9.71 (0.53)	10.85 (0.23)	8.94 (0.66)	10.62 (0.10)	8.35 (0.78)	10.76 (0.18)
24	11.60 (0.71)	10.74 (0.13)	10.59 (0.51)	10.62 (0.44)	8.32 (0.15)	9.85 (0.62)

Values are mean (*n* = 3) followed by (SE).

### 3.3. Prebiotic Effects

Shifts in *Bifidobacteria* and *Lactobacilli* were used to demonstrate the prebiotic effects measured in the batch culture fermentation system. For both genera of analysis, all three samples had similar baseline concentrations. At 12 h WD was slightly more bifidogenic than PHGG (*p* = 0.052), and also at 24 h (*p* = 0.043) (Table 3). Changes in *Lactobacilli* at 12 and 24 h for both WD and PHGG show that both fibers demonstrate lactogenic prebiotic properties. Based on fermentation kinetics, PHGG is more rapidly fermented than WD, and both fibers show prebiotic effects, based on changes in *Bifidobacteria* and *Lactobacilli*, as early as 12 h.

**Table 3:** Alterations in the Microbiota Concentration in Batch Culture Fermentation with WD and PHGG after 0, 12 and 24 h of Fermentation with Human Fecal Inoculum.

Time (h)	*Bifidobacteria* (CFU log_10_/mL)			*Lactobacilli* (CFU log_10_/mL)		
	WD	PHGG	Blank	WD	PHGG	Blank
0	9.24 (0.03) a	9.14 (0.07) a	9.08 (0.00) a	10.39 (0.04) a	10.40 (0.09) a	10.35 (0.02) a
12	9.50 (0.03) b	9.30 (0.04) b	9.05 (0.06) a	10.79 (0.04) b	10.86 (0.07) b	10.35 (0.02) a
24	9.41 (0.07) b	9.27 (0.04) c	8.96 (0.07) a	10.76 (0.09) b	10.68 (0.04) b	10.23 (0.03) a

Notes: Values displayed are mean (*n* = 2) (standard error); Values with different letters are statistically different from each other within rows of three columns displaying data for each respective genus (*p* < 0.05).

## 4. Discussion

The human gut microflora is a diverse population, with many different genera of bacteria having very different influences on the host. Both *Lactobacillus* and *Bifidobacterium* are generally accepted as two beneficial genera of bacteria, and contribute to a myriad of health benefits to the host [4]. Mechanisms underlying these benefits are thought to be through modulating the immune response and antagonizing pathogens, either by production of antimicrobial compounds or through competition for mucosal binding sites [16]. Additionally, these gut microbes facilitate nutrient and energy extraction from the diet [17]. Therefore, increasing dietary fiber intake while modulating the gut microbiota through supplementation with dietary fibers with prebiotic activity may aid in promoting the gut health of the host. Long-term studies have shown that fiber supplementation can alter the makeup of the intestinal microflora with soluble fibers [18], while the purpose of this study was to evaluate the early prebiotic effects employing an *in vitro* model.

Quantitative PCR analysis of WD and PHGG display that both are prebiotic dietary fibers, with both fibers displaying bifidogenic and lactogenic properties as early as 12 h when compared to baseline measurements. Previous studies have also shown bifidogenic and lactogenic properties for WD and PHGG at 24 h, while this is the first study to show rapid prebiotic activity at 12 h. Between fibers, WD is statistically more bifidogenic at 24 h (*p* = 0.043) and shows a statistical trend (*p* = 0.052) to be more bifidogenic at 12 h when compared to PHGG, although PHGG still shows bifidogenic and lactogenic growth at both 12 and 24 h. WD has also been shown to increase other genera of beneficial bacteria, including non-pathogenic *Clostridium* and *Roseburia*, in similar models [19].

Secondary measurements of fermentation kinetics, including change in pH, total gas production and development of SCFAs were also key components to modeling the mechanism of action for these fibers. For WD and PHGG, pH decreased from baseline until the 24 h time point. It is postulated that a decrease in gut pH allows for more efficient absorption of specific minerals [20,21], and may provide protective effects in the colon.

Both fibers did not produce detectable amounts of gas until 8 h, and for the 12 and 24 h measurements, PHGG produced significantly more total gas. Measurement of gas production is of interest as an indirect measurement of fiber fermentation. Work in our laboratory found that production of breath hydrogen and methane was a poor indicator of apparent digestion of fiber [22]. Acute tests of breath hydrogen production find that fibers that are fermented quickly and extensively, such as inulin, produce the most breath hydrogen [23,24]. In an *in vitro* system like the one described in this paper, differences among fibers on gas production should be useful indicators of the rate and extent of fermentation in the human gut.

Acetate, propionate and butyrate are commonly measured SCFAs and are representative end-products of colonic fermentation and are typically influenced by both microbes present and substrates utilized. Acetate is commonly metabolized by muscles for energy [25], propionate used as a gluconeogenic substrate [26] and butyrate used as an oxidative fuel for colonocytes [27]. Production of these acidic metabolites also promotes a decrease in colonic pH [28], which promotes the growth of many lactic acid bacteria that thrive in more acidic environments. Clinical studies have also shown that this decrease in pH promotes growth of beneficial bacteria such as *Bacteroides* and inhibits growth of *Clostridum perfringens* [29]. Based on secondary fermentation measurements, PHGG is more rapidly fermented than WD.

Because of the rapid formation and absorption of SCFAs *in vivo*, *in vitro* models are typically employed to accurately understand the kinetics of colonic fermentation. Although pooled fecal homogenates were used in this study, stimulation of bifidogenic and lactogenic bacteria are consistent with other clinical studies, demonstrating the prebiotic effects of WD and PHGG. The current study demonstrates that WD and PHGG act as prebiotic fibers and the prebiotic changes can occur as early as 12 h. Future research should be conducted using individual fecal sampling to analyze specie-specific stimulation of WD and PHGG, and doing so will allow for accurate comparisons between individuals in the population and help us to better understand the relationship between fiber consumption, the host’s microbiome and overall digestive health.
